# The Voluntary Hospitals

**Published:** 1921-10

**Authors:** E. W. Morris

**Affiliations:** C.B.E. (House Governor, London Hospital)


### THE VOLUNTARY HOSPITALS.

By E. W. Morris, C.B.E. (House Governor, London Hospital).

That the voluntary hospitals are in very great financial difficulties has already become "stale news," and consequently it is not easy to rouse public opinion as to the gravity of the situation.

We cannot blame the public. We have all met so many crises in the last seven years that one more makes very little difference. During the War there was a crisis every day, and since the War there have been almost as many and as serious. Before the importance of the Irish question, and the labour question, and the land question, and the unemployment question, and the ever-present question of making both ends meet, the hospital question sinks into obscurity. But it must not be allowed to sink into obscurity, and the public must be made to see that if the health of the country is in jeopardy, everything that depends on health, public and individual, will be in jeopardy too. I venture to state that I am not begging the question when I assert that the health of the country is so bound up with the work of the hospitals that if the one is in danger the other will be affected.

### What the Present Crisis May Mean.

In the hospital crisis through which we are passing there are two outstanding features which should claim attention. First then. The present trouble is not individual and local, but is widespread. It is not that some one hospital is in difficulty, nor even a group of hospitals in some area, but that all the hospitals in London and the provinces are in trouble. It is that the voluntary hospital system is in danger. It is not a question of some gallant ship being in peril from fog and rock and sandbank, but that the whole fleet is passing through a terrific storm with terrors none have known before, and no one can foretell what to-morrow may bring. And the second important feature of the present crisis is this, that if the hospitals fail, a great deal more will fail also. The fleet we have seen fighting the storm is fighting for much more than its own life. Its destruction will leave fair lands and happy homesteads to the mercy of the enemy.

It must be understood that the hospitals have long outgrown the purpose for which they were originally founded. They are no longer simply refuges for poor sick people. They have become centres for research and education as well as for treatment. Think of it. Every doctor was trained at one of them, and every nurse. The high code of honour of the hospital influences them through their professional career. When the scourges of the last century met their conqueror, the champion stepped out from the gates of a hospital. Diphtheria, and smallpox, and typhus, and surgical sepsis have a mighty respect for the hospitals if we have not. I like to think of the hospitals keeping watch and ward over London. I hope I am not divulging what I ought not when I state that not once or twice has my own hospital, the "London," kept watch while London slept safe at nights. We are near the docks. The telephone rings. "Do not draw attention to it, but we have unfortunately found some plague rats at the docks. Probably by ship from———. We are using every possible precaution, but—in case—you understand? The 'London' will be ready?" "The London will be ready."

And so, you see, the work of the hospitals has extended. What is going on in their wards and their laboratories closely affects the lives of those who will never enter them. Their work becomes more and more preventive, and therefore more closely linked up with other movements which have for their aim the improvement of and protection of health. They have ceased long since to be isolated islands of charity.

### Ruin Follows a Great Conception.

Now the reasons which have brought about the financial ruin of the hospitals are two. First and obviously, the War and the increased cost of nearly everything which a hospital needs, including the cost of service, salaries, and wages; the cost of material is falling, but the cost of service is not likely to fall.

And the second reason is, the growing complexity and cost of medical treatment following expanding knowledge of disease. When a business-house spends capital on the erection of new buildings, it invests in an earning department; when a hospital spends money upon a new laboratory, or wing, it spends money on a new spending department. Therefore when the outlook of a hospital changed from being a place where sick people lay waiting to get well, to being a place where sickness was studied and resisted, it soon found that it had adopted a very costly conception of its duty. Hardly a year but some new spending department had to be opened—a bacteriological laboratory, a bio-chemical laboratory, aseptic operating system, electrical departments, finsen light department, röntgen-ray departments, inoculation departments, light baths, physical exercises, and others. And each of these departments, filled with the same zeal as their mother, began to grow and develop and branch out. Indeed, the hospital, having adopted the new vision as to its responsibilities, finds there is no limit to the calls for improvement and greater efficiency and costly equipment and highly technical service. And so, while to the casual passer-by the great building stood simply as a place of shelter for the sick poor, it was in reality a great research centre working for the benefit of the non-sick, rich and poor alike. That great conception, an honourable one, has brought about more than anything else the ruin of the fine old hospitals. They have tried to do too much under the old conditions.

### The Failure of Charity.

We had better face it. Charity alone will not pay for the whole work of a modern hospital.

**The Voluntary Hospitals—(*cont.*).**

Charity will do all that the hospital was founded to do—treatment of the sick poor within its walls who cannot pay for the cost of their cure. But charity alone is failing to pay for costly research, which ultimately leads to discoveries which lay bare the life history of some group of allied diseases, and at last proclaims, "These things shall destroy no more for ever." And yet every one of us is safer, and so are our children, since that "All Clear" bugle sounded.

The cost per bed per week at my hospital has increased in ten years from £2 to £5. Every penny of that increase is well spent—it is "lives o' men." "Charity" brought in the two; it will not bring in the five.

What is to be done? Income must be increased somehow. It is becoming more and more usual for the hospitals to endeavour to increase income by asking patients to contribute part of the cost of their treatment. The amount asked for varies—some are asked for one guinea a week, some for two. Before long every hospital will be forced to take some such action. The only alternative is to reduce work and close beds.

Those hospitals which have inaugurated a part-payment system very soon found that when the wage-earner is the patient it was impossible to enforce the payment for the reason that wages had stopped. They therefore looked about for some method of getting over this difficulty. Some system of insurance was the obvious remedy—payment in very small amounts when well to insure free treatment when ill. Some of the hospitals began to organise group insurance in this way. A big factory, such as Messrs. Bryant & May's, or The Tunnel Cement Works would come to a friendly arrangement with the hospital that in consideration of certain weekly payments free service would be given to the employees when necessary. The hospitals were at a disadvantage in making these arrangements, for it was not possible to foresee what was likely to be the incidence of "hospital sickness" in a factory of average healthy employees. We have assumed that one in fifty of such would need to be admitted to the hospital during the year. The average cost of each in-patient admitted is £9 12s. 3d. (an average residence of fourteen days). If the fifty workmen give 2d. a week and the employers give 1d., a sum of £32 10s. per annum is handed to the hospital, which pays for the cost of the one admitted, and possibly a visit to a convalescent home afterwards; any out-patient treatment necessary for the fifty; and leaves a margin for cost of treating "dependants."

An Experimental Scheme.

This insurance scheme has been improved upon in the Sussex Scheme, which three of the hospitals of London are adopting experimentally for one year. I need not go into minute details, but an outline of the scheme is: That for certain annual payments, viz. £1 for a single person with income not over £250; £1 10s. for married couple without children, and income not over £400; £2 for married couple, with children, with income not over £500, the following advantages are offered by the hospital *on the request of the patient's doctor* (which is an essential part of the agreement):—

Consultations at the hospital, or, if necessary, at the bedside of the patient within certain distances (beyond these distances the only cost will be a reduced travelling charge for the consultant).

Admission to hospital beds, if necessary, after consultation.

A home nursing service.

An ambulance service.

Laboratory, *x*-ray, dental, massage, electrical services.

Radium treatment.

The great advantages of this scheme are that it brings the staff and equipment of the hospital to the aid of the general practitioner, and thus benefits the middle-class patient who is at present debarred from the advantages of hospital treatment.

The scheme is being organised by a committee of well-known and experienced men, as follows: The Hon. Sir Arthur Stanley, G.B.E., &c., The Lord Dawson of Penn, G.C.V.O., &c., Sir Alan G. Anderson, K.B.E., W. McAdam Eccles, F.R.C.S., J. F. Gordon Dill, Esq., M.D. (Hon. Secretary). All particulars may be obtained from Dr. Gordon Dill, Hon. Secretary, 77 Cambridge Terrace, W. 2.

The hospitals are not organising the scheme, but have been asked to co-operate, and are prepared to honour the bond.

**The Official Definition of a Hospital.**

We understand that the Voluntary Hospitals' Commission has adopted the following definition of a voluntary hospital: "An institution (other than an out-patient dispensary) managed by a responsible committee, and wholly or mainly supported from voluntary sources (including income derived from endowments or investments), the object of which is to provide medical or surgical treatment of a curative character; an auxiliary institution (such as a convalescent home), being eligible for assistance only in so far as it increases the facilities of hospitals from which it receives patients."

**Hospital Propaganda at Canterbury.**

To raise the necessary extra £8,000 a year for the Kent and Canterbury Hospital, a scheme has been devised which has begun to bear fruit. Under a Hospital Propaganda Committee, East Kent, including Canterbury, has been divided into about 160 districts, on parochial lines, wherein a box-collection scheme is being organised. So far sixty-three districts have been started with 9,000 boxes. The scheme has raised nearly £1,300 in six months. If it can be extended throughout the area secured by the hospital the extra income will be raised.

**Index to "The Hospital."**

The Index and Title Page to Vol. LXX., which was completed with the issue of September 24, 1921, are ready and will be sent to subscribers on application.

